# Complementary effects of probiotics and stimbiotics inclusion on growth performance, protein utility, serum metabolites and behavioural observations in broiler chickens exposed to cyclic heat stress

**DOI:** 10.1016/j.psj.2025.105606

**Published:** 2025-07-26

**Authors:** RM. Mokonyama, U. Marume, Ghaneshree Moonsamy

**Affiliations:** a Department of Animal Science, School of Agricultural Sciences, Faculty of Agriculture, Science and Technology, North- West University, P. Bag X 2046, Mmabatho, South Africa; b Food Security and Safety Niche area, Faculty of Agriculture, Science and Technology, North- West University, P. Bag X 2046, Mmabatho 2735, South Africa; c Council for Scientific and Industrial Research, Pretoria, ZA, South Africa

**Keywords:** Growth rate, Protein utilization, Serum electrolytes, Welfare

## Abstract

The study investigated the complementary effects of probiotics and stimbiotics inclusion on growth performance, serum metabolites and behavioural responses in broiler chickens exposed to cyclic heat stress. Six hundred Cobb500 day-old-chicks were allocated to five dietary treatments: Standard broiler diet, with no inclusion of AGPs (**NegControl**); Standard broiler diet, with inclusion of AGPs (**PosControl**); Standard broiler diet + 0.01 % probiotics (**Prob**); Standard broiler diet + 0.01 % stimbiotics (**Stim**) and Standard broiler diet + 0.01 % probiotics and 0.01 % stimbiotics) (**ProbStim**). Each dietary treatment was replicated 8 times, with a pen holding 15 birds as the experimental unit. The experimental trial was conducted over three feeding phases: starter phase (day 1- 14), grower phase (day 15- 28) and finisher phase (day 29- 42). The birds were subjected to heat stress in the grower phase and finisher phase for 3 h (11:00- 14:00 h). Diet had no effect on ADFI in the starter, grower and finisher phase. Similarly, diet had no effect on ADG and FCR in both grower and finisher phase. The cumulative weight gain was consistently low in broiler chickens fed the Stim diet throughout the feeding phases, while those fed Prob diet had higher weights in week 2 and to 6. Dietary treatments did not affect protein consumed, protein efficiency ratio, specific growth rate and growth efficiency across all the feeding phases. Total cholesterol and triglyceride concentrations were significantly affected by diet (*P* < 0.05) with broiler chickens fed PosControl in non- stressed environment having the highest total cholesterol (3.90± 0.18) and triglyceride level (1.77± 0.12). With regards to behavioral response to heat stress induced discomfort, broilers fed PosControl (2.33±0.26) and Prob (2.33±0.26) exhibited increased pecking feed activity and increased tendencies of seeking cooler areas. Overall, the birds did not show signs of severe stress. In conclusion, Probiotics and Stimbiotics could be used in combination to replace of AGPs without negatively affecting productivity in broilers.

## Introduction

The global demand for poultry products (eggs and meat) has been on a steep incline over the years, with the increase in global population (Heise et al., 2015). According to Gogia et al. (2018) and Ayu Shazwani and Rabeta (2020), the current global human population is projected to increase from 7.6 billion to 8.6 billion in 2030, 9.8 billion in 2050 and 11.2 billion in 2100. This increase is not being matched by a corresponding increase in food supply, posing a serious threat to food and nutrition security globally, particularly in the resource poor countries. The increase in population is generally linked to an increase in demand for food including of animal origin (Mtolo et al., 2022). In animal food production, the broiler industry is currently playing a big role in offsetting some the challenges of inadequate animal protein supply for humans (Elmahdy et al., 2025; Reda et al., 2024). Optimisation of broiler meat production has been quite significant over the years, mainly through advanced genetic improvements over a short period and the efficient use of antibiotic growth promoters **(AGPs)** to improve feed efficiency, promote resilience to heat stress and protect the birds against common poultry infections (Saracila et al., 2021; Wu et al., 2020). Nevertheless. the increased public health concern over the overuse and misuse of AGPs, has resulted in an associated increased demand for antibiotic free and organic products (Mohammadigheisar et al., 2019; Quinto et al., 2019; Samad, 2022). Thus, the European Union and many developed nations have implemented a ban the use of antibiotic growth promoters in food producing animals (Anadón et al., 2018; Tang et al., 2017), stimulating intense efforts to explore alternatives to antibiotics with equal effectiveness in enhancing broiler production (Elmahdy et al., 2025; Reda et al., 2024). Nutritional interventions, that include the inclusion of probiotics and stimbiotics in place of conventional antibiotics are currently being explored.

The use of novel feed additives such as probiotics known as “direct- fed microbes” have potential to improve broiler performance while alleviating the negative effect of heat stress which is also posing a significant challenge to broiler production in the tropics and subtropics (Jiang et al., 2021; Vimon et al., 2023). Probiotics are reported to modulate the gut composition by increasing population of beneficial microbes and inhibiting growth of harmful pathogens, while enhancing nutrients utilization immune development and overall productivity (Feseha et al., 2021; Wu et al., 2023). On the other hand, stimbiotics, which are non-digestible but highly fermentable nutritional additives, are currently emerging as possible and promising approach to improve nutrient digestion and utilisation, and overall animal performances (Cho et al., 2020; Luo et al., 2021). According to Morgan et al. (2021) and Song et al. (2022) stimbiotics are non-nutritive, yet prompt and encourage growth and activity of beneficial microbes, with subsequently enhancement of hind gut fermentation and absorption of nutrients in monogastric animals.

A lot of work has been done on the inclusion of various probiotics, mostly from international sources on broiler performance. Currently the CSIR (SA) is involved in the development of locally produced *Bacillus subtillis* based probiotics that complements the current environments and broiler production systems that is being applied in South Africa. Therefore, it is prudent to evaluate the effectiveness of locally produced probiotics in broiler production systems. Their inclusion in broiler diets together with stimbiotics may stimulate synergism in improving growth performance in broilers while offsetting the effects of heat stress. This study therefore assessed the effect of inclusion of locally produced *B. subtilis* based probiotics and stimbiotics on growth performance, protein utility, serum metabolites and behavioural patterns in broiler chickens exposed to cyclic heat stress.

## Material and methods

### Study site and ethical considerations

The study was conducted at the North-West University poultry unit in the North- West province of South Africa at GPS coordinates of 25°28´00˝S and 25°28´0˝E. The poultry unit is 1290 m above the sea level and receives average annual rainfall ranging from 300 to 600 mm annually. The area’s winter season temperature ranges from 2°C to 25°C and summer temperature ranges from 18°C to 29°C. All protocols used in the study were evaluated and approved by the NWU-ANIMPROD REC of the North-West University (SA), and the ethical clearance number (NWU-00802-24-A5) was granted.

### Sourcing of feed ingredients

The locally produced *Bacillus* based probiotic was obtained from the CSIR (SA), while the stimbiotic was obtained from Avipharm (Pvt, Ltd) in partnership with AB Vista (United Kingdom). Commercial broiler diets and antibiotic growth promoters were purchased from Simplegrow (Pvt) Ltd and Nutroteq (Pvt) Ltd. The CSIR institute is involved in the production of environmentally adapted probiotics for use in animal production and for human health.

### Animals, experimental design, and diet formulation

A total of 600 Cobb500 day-old- chicks were purchased from Chicken Ranch (PYT) Ltd Pretoria, Gauteng, South Africa. The birds were group weighed upon arrival and allocated randomly and evenly to five dietary treatments spread in 40 pens. The pens measuring 1.5 × 1.5 m (each) were designed to meet the welfare and optimum production of broiler chicks. Each pen housed 15 birds, and each dietary treatment were replicated 8 times with the pens considered as the experimental unit arranged in a completely randomised design **(CRD)**. The pen houses were equipped with a single feeders and drinkers. Feed and clean drinking water were offered *ad libitum* for the 42-day experimental period. The experimental diets were formulated to meet the nutritional requirements of broiler chicken from starter to finisher phase. The feed additives (probiotic and stimbiotic) used in this study replaced conventional antibiotic growth promoter (AGPs) in the diets and the dietary treatments were formulated as follows: T1: Standard broiler diet, with no inclusion of AGPs (NegControl); T2: Standard broiler diet, with inclusion of AGPs (PosControl); T3: Standard broiler diet + 0.01 % probiotics (Prob); T4: Standard broiler diet + 0.01 % stimbiotics (Stim); T5: Standard broiler diet + 0.01 % (probiotics+ stimbiotics) (ProbStim). The inclusion of the probiotics and stimbiotics was set at 0.01 % of the diet, in accordance with the recommendations of the supplier and the available literature. The feed additives were included in pre-formulated diets free of any medication.

### Feeding, growth trial, management of animals and ethical clearance

On arrival, the birds received stress pack for first three consecutive days for acclimatisation to the environment. The birds were phase-fed, beginning with provision of starter feed ration (day 1 to day 14), then grower phase (day 15- day 28) and finisher phase (day 29- day 42). The experiment was conducted under optimum temperature and humidity and the birds were regularly monitored to check for any mortalities. From day 15, at the start of the growth phase onwards the birds were subjected to cyclic heat stress (stressor) at temperature that ranges between (30-35°C) at grower phase (on day 27) and finisher phase (day 41) for 3 hrs (11:00- 14:00) following the protocols of Mahammad et al. (2018). The subjection to heat stress was achieved through use of infra- red lights to increase the temperature of the poultry house and induce some inflammation or reaction phase that allowed for the evaluation of their responses to the inclusion of the probiotic and stimbiotic in diets.

### Growth performance and feed utilization

Ten chickens from each pen were randomly selected and weighed individually using TSW equipment weighing scales/Adam equipment at the beginning of the experiment to determine the initial body weight and weekly thereafter. Mortality was recorded daily. Body weight gain (**BWG**) was calculated on a weekly basis throughout the feeding trial. Daily feed intake was determined by measuring the difference between the feed given and the feed refusal every morning before feeding and was adjusted according to the recorded mortalities. The average daily feed (**ADFI**), average daily gain (**ADG**) and feed conversion ratio (**FCR**) per feeding phase were computed as follows:

$$Average\;daily\;feed\;intake\;\left(ADFI\right)=\frac{Feed\;intake\;\left(g\right)}{number\;of\;days\;\left(14\;days\right)}$$

$$Average\;daily\;gain\;\left(ADG\right)=\frac{Body\;weigt\;gain\;\left(g\right)}{numer\;of\;days\;\left(14\;days\right)}$$

$$Feed\;conversion\;ratio\;\left(FCR\right)=\frac{daily\;feed\;intake\;\left(g\right)}{daily\;weight\;gain\;\left(g\right)}$$

### Protein utilization and growth efficiency

The protein consumed (PC /bird) was computed by multiplying the amount of crude protein (**CP*d***) in the diet (g/kg DM consumed) and the total feed intake per feeding phase. Protein efficiency ratio (**PER** g/kg) was computed by dividing the total body weight gain by the protein consumed calculated previously, while specific growth rate (**SGR**) known as percentage of growth per feeding phase and growth efficiency (**GE)** was calculated as:

PC=FI×CPd

PER=BWG/PC

SGR=(Finalweight−initialweigt)/(initialweihgt)×100

GE=BWG/(Initialweight)

### Blood collection and analysis

At day 20 (before the subjection of the birds to heat stress) and day 41 (subjection of the birds to heat stress), 3 chickens from each pen were randomly selected and blood (approximately 2 - 4 ml) was drawn from the branchial vein using a needle and syringe and collected into 3 sterile tubes (yellow, grey and red) for analysis of clinical chemistry parameters, liver enzymes, pancreas and gastrointestinal, carbohydrates metabolism and lipids parameters (generally serum biochemistry). Serum was obtained through centrifuging blood at 1000 g for 15 minutes.

### Behavioural observation to heat stress

Heat stimulus was applied at 32 - 35°C for three hours (11:00 - 14:00) starting from grower phase (on day 27), and on the finisher phase (day 41). The heat stimulation was only done two times on a weekly basis (on grower and finisher phase). Behavioural heat stress markers were observed during heat stress on day 27 of the grower phase. Table 2. illustrate the estimated temperature profile for induced patterns of heat stress, with humidity index recordings. With regards to behavioural responses, observations were conducted manually on three randomly selected pens from each treatment. Chicks were observed based on the ethogram (Table 3) introduced by (Santos et al., 2015). The pen house was equipped with thermohydrometer to measure humidity and temperature. The normal or ideal comfort zone for broiler chickens ranges from 18 - 22°C, where one can anticipate an optimum metabolic activity, no heat stress behaviour such as panting and cold stress. However, on day 27 and 41, temperature was set above the chicks’ comfort zone between 32 - 35°C (ambient temperature), using infra- red lights. The birds were subjected to a higher environmental temperature (heat stress) than their thermoneutral temperature and tried to dissipate excess heat produced inside the body, which is manifested by specific behavioural changes. During the heat stress phase, observations were made on day 27, from 11:00 to 14:00 hr, allowing consistent monitoring. Water intake was determined by measuring the difference between the initial water volume and the remaining water volume after heat stress stimulation. The behavioural such as characteristic signs of panting, feather pecking, wing spread, walking and standing were observed from the pens at 1.5 m to avoid interruption. A scale from 0 – 4 was used to quantify the intensity of each behaviour observed during each interval throughout the heat stress exposure. The scoring system was planned as:

- 1: Normal response/ no stress
- 3: High stress level
- 4- 5: Severe stress level

Average water intake was expressed as follows

$$Average\;daily\;water\;intake\;\left(ADWI\right)=\frac{water\;intake\;\left(l\right)}{number\;of\;days}$$

### Statistical analysis

The data on growth performance, protein utility, serum metabolites and behavioural observations were analysed using general linear model (GLM) procedure of SAS (2010). Data cumulative weight gain was measured on a weekly basis and was analysed using the mixed model procedure of SAS (2010) with the inclusion of initial weight as a covariate. The probability differences option of SAS (2010) was used to perform comparisons of the least square means, while contrasts (SAS, 2010) was used to determine the specific effects of the *Bacillus* based probiotic and stimbiotic and their interaction on different parameters.

The statistical models were as follows:

$$Y_{ij\;}=\;\mu \;+\;T_{i}\;+\;\varepsilon {\;}_{ij}$$

Where: Y_ij_ = observation (growth performance, protein utility, serum metabolites and behavioural observations), μ = population mean constant common to all observations, T_i_ = effect of diet, and ε_ij_ = random error term. The repeated measures model for analysis of cumulative weight gain included the effect in week of measurement and the diet *x* week interactions. For all tests, the level of significance will be set at (*P* < 0.05).

## Results

### Growth performance and feed utilization

**Fig. 1:**
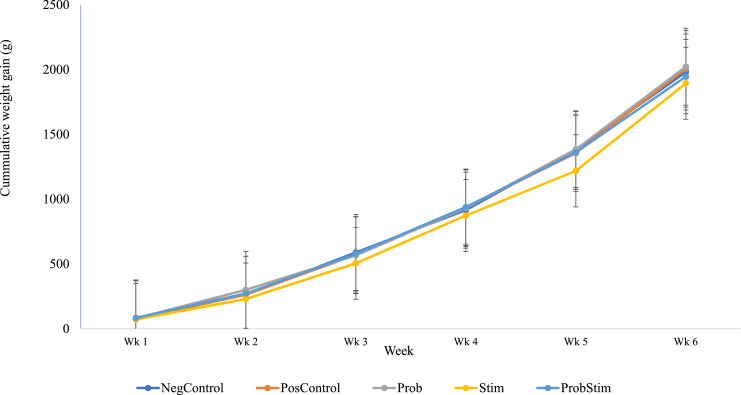
Complementary effect of probiotic and stimbiotic on cumulative weekly weight gains in broilers exposed to cyclic heat stress.

The complementary effect of probiotic and stimbiotic on growth performance parameters in broiler chickens exposed to cyclic heat stress are presented in Table 4. From the results, diet had no effect on ADFI in the starter, grower and finisher phase. Similarly, diet had no effect on ADG and FCR in the grower and finisher phase. However, significant differences (*P* < 0.05) were observed in ADG of broilers in the starter phase. Broiler chickens fed Prob diet (21.08 ± 0.98) had the highest (*P* < 0.05) ADG while those fed the Stim diet (16.37 ± 0.98) on its own had the lowest. Overall, no differences in ADFI, ADG and FCR were observed throughout the experiment. The cumulative weight gain of birds over the experimental period is presented in Fig. 1. From the results, there was a consistent increase in the body weight gain across treatments throughout the experiment. Notably, there were significant differences (*P* < 0.05) in cumulative weight gain of broilers across treatments at week 2, 3 and 5 of the experiment. The cumulative weight gain was, however, relatively low in the group fed stimbiotic compared to other dietary groups, while those fed probiotics showed marginally higher gain in week 2, coupled with a rapid gain from week 5 to week 6.

### Protein utilization and growth efficiency

The complementary effect of probiotics and stimbiotics in broilers exposed to cyclic heat stress on protein utilization and growth efficiency is presented in Table 5. As shown in the table, dietary treatments did not have any effect on protein consumed, protein efficiency ratio, specific growth rate and growth efficiency across all the feeding phases.

### Serum biochemical analysis

Table 6 shows complementary effect of probiotic and stimbiotic on serum electrolytes in broilers subjected to heat stress. As illustrated on the table, no dietary influences were observed on all serum electrolytes in broilers. Similarly, no dietary influence was observed in broilers on liver enzyme activity (Table 7). The results of serum metabolic markers in broiler chickens subjected to heat stress are presented in Table 8. From the results, no effects of diet on serum metabolic markers such as urea, creatinine and total bilirubin were evident. Nevertheless, the levels of total cholesterol and triglyceride were significantly influenced (*P* < 0.05) by the diet in non- stressed (normal) environment. Broiler chickens fed PosControl in non- stressed environment had the highest total cholesterol (3.90± 0.18) and total triglyceride level (1.77± 0.12) compared to all other treatments, with Stim having the lowest triglyceride content (1.00± 012).

### Behavioural responses

The complementary effect of probiotic and stimbiotic on behavioral observations in broilers exposed to cyclic heat stress is presented in Table 9. Diet had no effect on behavioral observations, apart from pecking feed, chicks seeking cool areas and resting (*P* < 0.05). From the results, it can be confirmed that broilers fed PosControl (2.33±0.26) and Prob (2.33±0.26) showed increased pecking of feed. Similarly, from the same dietary treatments, chickens sought cooling areas more than those fed other dietary treatments. Stim (3.00±0.26) diet produced closely matched results with PosControl (3.33±0.26) and Prob (3.33±0.26) in terms of seeking cool areas. Broiler chickens directly fed probiotic alone (Prob) (3.67±0.21) showed more resting effect than other treatments. Nonetheless, The inclusion of the probiotics and stimbiotics appeared to have indifferent effects on overall behavioral responses.

## Discussion

### Growth performance and feed utilization

In the poultry industry, it is crucial to find safe, sustainable and effective alternatives to antibiotic growth promoters that may offer wholesome, safe and healthy products (Reda et al., 2024; Tiseo et al., 2020). There is an increase concern on the continues use of antibiotics in meat production due to the emergence of residues and antibacterial resistance in the value food chain, (Dutta et al., 2019; Rahman et al., 2022). This study therefore assessed the inclusion of probiotics and stimbiotics in broiler diets as replacements for conventional growth promotants. In this study, the supplementation of a locally produced probiotic (*B. Subtillis*) in broiler chickens during starter phase led to an increase in ADG, followed by the combined inclusion of probiotic and stimbiotic. The average daily gain is a comprehensive metric that is used to measure the average increase in the body weight per day, reflecting chickens’ growth rate and efficiency (Lakamp et al., 2022). Previous studies have showed that the *B. subtilis* based probiotic can significantly promote the growth performance of broiler chickens (Bai et al., 2017; Mohamed et al., 2022; Qui et al., 2021). The findings of this study, therefore, align with the existing literature, demonstrating that probiotics and their combinations with stimbiotic may significantly enhances the ADG of broiler chickens (Pandey et al., 2022). The decreased ADG in broiler chickens fed negative control (diet with no AGPs), is a clear indication of the importance of growth enhancers in broiler diets. On their part, the stimbiotics function in promoting the hind gut fermentation that increase energy metabolising but with no influence of overall digestion and nutrient assimilation processes (Ren et al., 2023; Taheri et al., 2010). In the current study, probiotic increased ADFI and ADG during the starter period, while reducing the FCR in grower phase, and then improved the ADG and FCR in the finisher period. These results align with previous studies demonstrating positive effect of probiotics on gut health, immune functions and nutrient absorption. While the stimbiotic on its own increased the FCR, the improved ADG in broiler fed the ProbStim could be attributed by probiotics rapid action to promote eubiosis and modulation of immune system, complimented by stimbiotics’ role to stimulate growth of beneficial microbes and short- chain fatty acids (SCFAs) production, creating a more stable and conducive gut environment. According to Su et al. (2020) probiotics exhibit properties of desirably altering gut microbiome diversity and increase the release of digestive enzymes such as proteases, lipase and amylase, which in turn increases utilization and digestibility of nutrients while the synergistic effect of probiotic and stimbiotic may offers a promising effect to improving intestinal morphology and intestinal microflora population and additional energy provision due to increase hindgut fermentation. With regards to the cumulative weight gain, a general increase in the weight gain was observed throughout the study as expected (Gao et al., 2017; Mijena and Getiso, 2024).

### Protein utilization and growth efficiency

In the current study, protein utilization and growth efficiency measurements were not affected by the dietary treatments in all the feeding phases. The protein utilization of chickens depends on the gut function, and protein content in the diet which is closely associated with the feeding phases. On the other hand, protein efficiency ratio measures the value of protein in a diet as influenced by the feed additives and other factors (Imari et al., 2023). Dietary protein is critical in muscle development and maintenance and growth of animals (Vogtschmidt et al., 2021). Although not significantly different, results of the study appeared to show that birds fed the Prob and ProbStim diets obtained marginally higher protein consumed, protein efficiency ratio, specific growth rate and growth efficiency compared to other treatments. This suggests that probiotics and synergistic effect of probiotic and stimbiotic enhanced the utilization and digestibility of amino acids, supporting optimum growth, maintenance and development. The results of the study by Mousapoor et al. (2023) showed that supplementation of probiotics at 1000 g/ton increased feed, energy and protein intake in broiler chickens. Similarly, findings from Imari et al. (2023) indicates that the broiler chickens fed diet containing probiotics at 100 mg/kg and 150 mg/kg improved the protein efficiency ratio and protein retention in the starter phase. Based on this evidence, it can be concluded that supplementation of probiotics can significantly enhance productivity, protein efficiency ratio and absorption of nutrients in low- protein diet (Imari et al., 2023). Stimbiotic supplementation in the grower phase resulted in high specific growth rate and growth efficiency probably due to increased energy provision through hind gut fermentation that release volatile fatty acids. In this regard, probiotic and stimbiotic can effectively maximize and better growth performance and nutrient digestibility tailored to dietary protein levels and growth phase.

### Serum electrolytes, serum enzymes and serum metabolic markers

Previous studies on heat stress in broilers have reported that measuring the serum biochemical and serum electrolytes can be important in evaluating the resilience, management practices, health and nutritional status and organ function of animals in response to external stresses (Liu et al., 2024; Rouissi et al., 2021). The measurements of serum electrolytes (Na^+,^ *K*^+,^ Cl^-^ e.c.t), liver enzymes (lipase, amylase, aspartate transaminase, alanine transaminase and alkaline phosphate) as well as nutritional status (urea, creatinine, total bilirubin, total cholesterol and triglyceride) (Huang et al., 2018; Monov and Molodozhnikova, 2024; Saki et al., 2016) can be key is assessing the response of the birds to external inflammatory factors. From the results, all serum electrolytes, and enzymes, along with metabolic markers, were not affected by the diet during the periods of exposure to cyclic heat stress as observed in other studies (Greene et al., 2024). However, in non - exposure phase, diet significantly affected total cholesterol and triglyceride content. The positive control and ProbStim (combination) groups showed higher levels of total cholesterol and triglyceride level, while the total cholesterol and triglyceride level was low in broiler fed stimbiotics. Conversely, the supplementation of stimbiotic individually shows a potential improvement in lipid metabolism through hindgut fermentation resulting in the release of volatile fatty acids. On contrary, the results of the study by Humam et al. (2020) indicated that supplementation of probiotics and postbiotics significantly reduced the total cholesterol levels. However, there is a gap in literature with limited information on the complementary effect of probiotic and stimbiotic on serum electrolytes, serum enzymes and serum metabolic markers in broiler chickens subjected to heat stress.

### Behavioural responses

The use of feed additives has been widely used in animal production system as antioxidants, antidote, immunomodulators, antibacterial, antiviral, antifungal, gastrointestinal stimulants and heat stress tranquilizers (Ayalew et al., 2022). Heat stress in broilers chickens can result in numerous adverse effects on behavioural and physiological changes (Elmahdy et al., 2025). Heat stress reduces feed intake, increases panting, lethargy and water consumption, (Wasti et al., 2020). With regards to the physiological changes, heat stress increase cortisol level, suggesting an elevated stress, alters metabolic process, resulting in increased reactive oxygen species which causes oxidative stress (Belhadj Slimen et al., 2014; Bueno et al., 2017; Fang et al., 2023). Similarly, heat stress disturbs the role of liver enzymes, as an indicative of dysfunctional metabolic process (Araújo et al., 2019). The inclusion of probiotics and stimbiotics may be one strategy the alleviate the negative effects of heat stress on broilers.

The results of the study showed significant effect on exposure to heat stress on behavioral observations. The behavioral observation patterns in broilers fed PosControl and Prob showed increased tendencies in terms of pecking of feed. This may reflect a behavioral response to heat stress, although the trends could also be linked to an urge to feed or a desire for feeding. The birds fed from the same diets also were seeking more shade than those fed other dietary treatments, escaping from heat exposure to regulate body temperatures, essential to reduce thermal load and maintain a stable body temperature. While certain groups showed higher feed pecking, these behaviors were assessed in the context of heat stress. When integrated with other behavioral patterns including reduced activity or increased seeking of cool areas, this behavior correlated total higher stress score. The broiler chickens fed Prob showed more resting behavior compared to the other treatments. This could imply that probiotics have beneficial effects as an anti-stress stimulant for maintaining health and welfare of broilers under stressful conditions (Islam et al., 2024). Ringseis and Eder (2022) reported several mechanisms of probiotics alleviating heat stress such as modulating the gut microbiome leading to improved feed consumption and reducing stress- related effects. Probiotics may also reduce oxidative stress and improve hydration status, by improving antioxidants activity, accompanied by reducing cortisol level and improving metabolic function, as well as maintaining electrolytes balance (Musazadeh et al., 2023; Piqué et al., 2019). that the birds have striven to attempt to regulate their body temperature, in response to the heat. Furthermore, the results showed that broilers fed Prob- supplemented diet were resting more than the other chickens from the dietary groups suggesting energy conservation strategy in response to the heat stress, to ensure continuous availability for other activities.

## Conclusion

The study demonstrated that the inclusion of probiotics and stimbiotics, individually and in synergy improved ADG, serum metabolic markers and modulate behavioural response. Particularly probiotics and synergy of probiotics and stimbiotics could improve early growth rate in broilers exposed to heat stress. Notably, probiotics diet showed a pronounced effect on cumulative weight gain in week 2 and week 5 to 6. Additionally, probiotics and stimbiotics individually improved lipid metabolism process of broiler chickens, supported by SCFAs production and liberation of digestive enzymes, enhancing barrier functions increasing the abundance of beneficial microbes of broilers. The synergy of probiotics and stimbiotics, and stimbiotic alone was partially effective in ameliorating heat stress in broilers exposed to cyclic heat stress. This findings suggest that probiotics and stimbiotics could be useful substituent to AGPs, while potentially mitigating heat stress in broiler chickens, providing cost- effective broiler production.

Table 1

**Table 1:** Ingredient composition (kg) of the dietary treatments at starter, grower and finisher phase.

Dietary treatments															
			Starter					Grower					Finisher		
Ingredients	T1	T2	T3	T4	T5	T1	T2	T3	T4	T5	T1	T2	T3	T4	T5
Yellow Maize-Fine	56.8	56.8	56.8	56.8	56.8	157.2	157.2	157.2	157.2	157.2	171.4	171.4	171.4	171.4	171.4
Prime Gluten 60	1.06	1.06	1.06	1.06	1.06	4.06	4.06	4.06	4.06	4.06	2.86	2.86	2.86	2.86	2.86
Fullfat Soya	0.0	0.0	0.0	0.0	0.0	11.4	11.4	11.4	11.4	11.4	3.44	3.44	3.44	3.44	3.44
Soyabean oilcake	28.5	28.5	28.5	28.5	28.5	44.26	44.26	44.26	44.26	44.26	40.5	40.5	40.5	40.5	40.5
Limestone Powder	1.46	1.46	1.46	1.46	1.46	3.28	3.28	3.28	3.28	3.28	2.92	2.92	2.92	2.92	2.92
MCP/Mono Cal KK	0.82	0.82	0.82	0.82	0.82	1.62	1.62	1.62	1.62	1.62	1.12	1.12	1.12	1.12	1.12
Salt-Fine	0.3	0.3	0.3	0.3	0.3	0.74	0.74	0.74	0.74	0.74	0.74	0.74	0.74	0.74	0.74
Koeksoda	0.16	0.16	0.16	0.16	0.16	0.38	0.38	0.38	0.38	0.38	0.28	0.28	0.28	0.28	0.28
Choline Powder	0.06	0.06	0.06	0.06	0.06	0.16	0.16	0.16	0.16	0.16	0.16	0.16	0.16	0.16	0.16
Lysine	0.22	0.22	0.22	0.22	0.22	0.62	0.62	0.62	0.62	0.62	0.58	0.58	0.58	0.58	0.58
L-Threonine	0.04	0.04	0.04	0.04	0.04	0.1	0.1	0.1	0.1	0.1	0.06	0.06	0.06	0.06	0.06
Methionine	0.2	0.2	0.2	0.2	0.2	0.42	0.42	0.42	0.42	0.42	0.36	0.36	0.36	0.36	0.36
BrStr premix+ Phy	0.16	0.16	0.16	0.16	0.16	0.0	0.0	0.0	0.0	0.0	0.0	0.0	0.0	0.0	0.0
BrGr premix+ Phy	0.0	0.0	0.0	0.0	0.0	0.38	0.38	0.38	0.38	0.38	0.0	0.0	0.0	0.0	0.0
Brfin premix+ Phy	0.0	0.0	0.0	0.0	0.0	0.0	0.0	0.0	0.0	0.0	0.38	0.38	0.38	0.38	0.38
Salinomycin	0.0	0.04	0.0	0.0	0.0	0.0	0.04	0.0	0.0	0.0	0.0	0.04	0.0	0.0	0.0
Olaquindox	0.0	0.04	0.0	0.0	0.0	0.0	0.04	0.0	0.0	0.0	0.0	0.04	0.0	0.0	0.0
Probiotic	0.0	0.0	0.009	0.0	0.009	0.0	0.0	0.0225	0.0	0.0225	0.0	0.0	0.0225	0.0	0.0225
Stimbiotic	0.0	0.0	0.0	0.009	0.009	0.0	0.0	0.0	0.0225	0.0225	0.0	0.0	0.0	0.0225	0.0225

^1^Dietary treatments: T1 = *a* negative control consisting of a commercial broiler diet without antibiotic growth promoter; T2 = *a* positive control consisting of a commercial broiler diet with antibiotic growth promoter; T3 = commercial broiler diet + *Bacillus* probiotic; T4 = commercial broiler diet + stimbiotic; T5 = commercial broiler diet + *Bacillus* probiotic + stimbiotic; MCP= Mono calcium phosphate; BrStr premix+ Phy= Broiler starter with phytase; BrGr premix+ Phy= Broiler grower with phytase; Brfin premix+ Phy= Broiler finisher with phytase.

**Table 2:** Temperature-humidity index (THI) for induced patterns of heat stress.

Bird age		Temperature patterns (°C)			
	Predicted readings	Humidity	Observed readings		Humidity
Behavioural observations Grower (day 27)	32-35	40-60 %	31.6		22 %
Blood collection Finisher (day 41)	32-35	40-60 %	33.0		22 %

The temperature profile presents a cyclic heat stress conditions with temperatures increasing from 11:00 to 14:00 and decrease after heat stimulation.

**Table 3:** An ethogram used to systematically observe and analyze the behavior and performance of broilers exposed to cyclic heat stress.

Type of behavior	Description/Definition
Pecking at feed	Observing the broilers pecking at their feed to assess their appetite and feeding behavior.
Drinking water	Monitoring the broilers’ water intake to evaluate their hydration levels and overall health.
Stretching	Elongation of wings and legs out- backward individually in a standing position
Panting	Monitoring panting behavior, which is a common response to heat stress in broilers.
Seeking shade or cool areas	Noting the broilers’ preference for shaded or cooler areas, indicating their thermoregulatory behavior.
Resting	Monitoring periods of rest or inactivity to evaluate the broilers’ stress levels and comfort.
Spreading wings	Observing broilers spreading their wings to release body heat and regulate temperature.

**Table 4:** Complementary effect of probiotic and stimbiotic on growth performance in broilers exposed to cyclic heat stress.

Dietary treatments							
Parameters	NegControl	PosControl	Prob	Stim	ProbStim	SEM	*P- value*
*Starter phase*							
ADFI (g)	41.62	42.29	43.04	40.75	42.81	1.35	NS
ADG (g/d/bird)	18.91^b^	18.81^b^	21.08^d^	16.37^a^	19.40^c^	0.98	*P* < 0.05
FCR	2.21	2.25	2.09	2.55	2.21	0.11	NS
*Grower phase*							
ADFI (g)	108.26	101.24	105.16	107.47	104.10	4.06	NS
ADG (g/d/bird)	37.38	38.80	36.09	39.91	36.14	2.04	NS
FCR	2.91	2.67	2.98	2.77	2.93	0.17	NS
*Finisher phase*							
ADFI (g)	161.24	162.11	160.54	172.14	159.42	7.95	NS
ADG (g/d/bird)	67.91	68.49	70.11	68.57	64.24	3.19	NS
FCR	2.43	2.37	2.30	2.54	2.48	0.12	NS

^a^.

^b^.

c Means in the same row with different superscripts are significantly different (*P* < 0.05); Dietary treatment: NegControl: commercial broiler diet without AGPs; PosControl: commercial broiler diet with AGPs; Prob; experimental broiler diet with 0.01 % probiotic; Stim: experimental broiler diet with 0.01 % stimbiotic; ProbStim: experimental broiler diet with 0.01 % combination of probiotic and stimbiotic; SEM: Standard Error Mean.

**Table 5:** Complementary effect of probiotic and stimbiotic on protein utilization efficiency in broilers exposed to cyclic heat stress.

Dietary treatments							
Parameters	NegControl	PosControl	Prob	Stim	ProbStim	SEM	*P- value*
*Starter phase*							
PC (g)	8.65	8.79	8.94	8.47	8.90	0.28	NS
PER	2.19	2.15	2.34	1.96	2.19	0.09	NS
SGR (% day^-1^)	15.14	14.84	15.56	14.49	15.52	0.41	NS
GE	0.53	0.50	0.57	0.49	0.56	0.03	NS
*Grower phase*							
PC (g)	19.49	18.22	18.93	19.34	18.90	0.73	NS
PER	1.94	2.15	1.91	2.08	1.93	0.12	NS
SGR (% day^-1^)	6.62	6.10	6.33	6.71	6.53	0.33	NS
GE	0.11	0.12	0.13	0.13	0.11	0.01	NS
*Finisher phase*							
PC (g)	25.80	25.94	25.69	27.54	25.51	1.27	NS
PER	2.64	2.64	2.77	2.54	2.56	0.14	NS
SGR (% day^-1^)	4.76	4.60	4.80	5.04	4.54	0.19	NS
GE	0.07	0.07	0.07	0.08	0.07	0.004	NS

Dietary treatment: explained in Table 3.1; PC; Protein consumed; PER; Protein efficiency ratio; SGR; Specific growth rate; GE; Growth efficiency; SEM: Standard Error Mean.

**Table 6:** Complementary effect of probiotic and stimbiotic on serum electrolytes (mmol/L) in broiler chickens.

Dietary treatments							
Parameters	NegControl	PosControl	Prob	Stim	ProbStim	SEM	*P- value*
*First collection (non- stress)*							
Sodium	150.67	150.67	151.00	151.00	148.00	1.31	NS
Potassium	5.10	6.30	5.67	5.97	5.13	0.64	NS
Chloride	110.33	110.33	110.33	112.00	109.67	1.01	NS
Calcium	2.35	2.45	2.40	2.34	2.36	0.074	NS
Phosphate	2.31	2.59	2.46	2.52	2.43	0.18	NS
Total carbon dioxide	21.67	21.67	23.67	19.33	25.00	1.83	NS
*Second collection (heat stress)*							
Sodium	148.33	148.33	147.33	149.00	150.33	1.32	NS
Potassium	5.43	5.07	5.80	5.10	5.33	0.64	NS
Chloride	110.33	108.67	110.67	110.00	109.67	1.16	NS
Calcium	2.41	2.48	2.35	2.36	2.60	0.06	NS
Phosphate	2.54	2.21	2.32	2.23	2.83	0.24	NS
Total carbon dioxide	24.67	26.33	24.67	25.33	25.33	1.17	NS

Dietary treatment: NegControl: commercial broiler diet without AGPs; PosControl: commercial broiler diet with AGPs; Prob; experimental broiler diet with 0.01 % probiotic; Stim: experimental broiler diet with 0.01 % stimbiotic; ProbStim: experimental broiler diet with 0.01 % combination of probiotic and stimbiotic; SEM: Standard Error Mean.

**Table 7:** Complementary effect of probiotic and stimbiotic on liver enzymes (U/L) parameters in broiler chickens.

Dietary treatments							
Parameters	NegControl	PosControl	Prob	Stim	ProbStim	SEM	*P- value*
*Before heat stress exposure*							
Lipase	81.67	85.33	41.33	53.67	87.67	27.94	NS
Amylase	1394.67	915.00	768.67	1207.67	3854.33	1401.66	NS
Aspartate transaminase	197.33	226.67	190.67	204.33	205.33	12.76	NS
Alanine transaminase	3.00	3.67	3.33	3.00	3.00	0.33	NS
Alkaline phosphate	38780.00	36520.67	20876.67	23670.67	33586.67	8974.28	NS
*After heat stress exposure*							
Lipase	24.67	16.33	28.67	25.67	23.33	6.07	NS
Amylase	882.00	421.00	579.33	362.67	441.67	148.07	NS
Aspartate transaminase	290.67	220.67	240.33	231.00	321.67	50.16	NS
Alanine transaminase	4.33	3.00	4.00	3.00	3.00	0.75	NS
Alkaline phosphate	5284.00	3644.67	7378.00	9832.33	5890.00	2204.14	NS

Dietary treatment: NegControl: commercial broiler diet without AGPs; PosControl: commercial broiler diet with AGPs; Prob; experimental broiler diet with 0.01 % probiotic; Stimb: experimental broiler diet with 0.01 % stimbiotic; ProbStim: experimental broiler diet with 0.01 % combination of probiotic and stimbiotic; SEM: Standard Error Mean.

**Table 8:** Complementary effect of probiotic and stimbiotic on serum metabolic markers in broiler chickens.

Dietary treatments							
Parameters	NegControl	PosControl	Prob	Stim	ProbStim	SEM	*P- value*
*Before heat stress exposure*							
Urea (mmol/L)	0.80	0.80	0.80	0.83	0.800	0.01	NS
Creatinine (mmol/L)	5.33	5.00	5.00	5.33	5.00	0.21	NS
Total bilirubin (Umol/L)	4.00	5.00	3.67	4.00	3.67	0.33	NS
Total cholesterol (mmol/L)	3.20^a^	3.90^b^	3.37^ab^	3.19^a^	3.87^b^	0.18	*P* < 0.05
Triglyceride (mmol/L)	1.33^b^	1.77^c^	1.23^ab^	1.00^a^	1.33^b^	0.12	*P* < 0.05
*After heat stress exposure*							
Urea (mmol/L)	0.83	0.80	0.83	0.80	0.80	0.02	NS
Creatinine (mmol/L)	5.33	5.33	6.00	5.00	5.33	0.52	NS
Total bilirubin (Umol/L)	5.67	5.00	5.67	4.67	4.67	0.83	NS
Total cholesterol (mmol/L)	3.17	2.60	3.47	3.23	3.27	0.40	NS
Triglyceride (mmol/L)	0.97	4.17	0.47	0.53	0.57	1.58	NS

^a^.

^b^.

c Means in the same row with different superscript are significantly different (*P* < 0.05); Dietary treatment: NegControl: commercial broiler diet without AGPs; PosControl: commercial broiler diet with AGPs; Prob; experimental broiler diet with 0.01 % probiotic; Stim: experimental broiler diet with 0.01 % stimbiotic; ProbStim: experimental broiler diet with 0.01 % combination of probiotic and stimbiotic; SEM: Standard Error Mean.

**Table 9:** Complementary effect of probiotic and stimbiotic on behavioral observations in broilers exposed to cyclic heat stress.

Dietary treatments							
Parameters	NegControl	PosControl	Prob	Stim	ProbStim	SEM	*P- value*
Peck feed	1.00^a^	2.33^c^	2.33^c^	1.67^b^	1.00^a^	0.26	*P* < 0.05
Stretching	0.67	1.33	1.67	1.33	2.00	0.30	NS
Panting	0.33	1.00	1.00	1.33	0.33	0.26	NS
Resting	2.33^a^	3.00^c^	3.67^cd^	2.00^b^	3.00^c^	0.21	*P* < 0.05
Spread wings	1.00	1.67	0.33	1.00	1.00	0.33	NS
Seek cool area	2.00^a^	3.33^bc^	3.33^bc^	3.00^c^	2.33^b^	0.26	*P* < 0.05
Average water intake (l)	0.09	0.06	0.06	0.07	0.07	0.01	NS

a, b, c Means in the same row with different superscript are significantly different (*P* < 0.05); Dietary treatment: NegControl: commercial broiler diet without AGPs; PosControl: commercial broiler diet with AGPs; Prob; experimental broiler diet with 0.01 % probiotic; Stim: experimental broiler diet with 0.01 % stimbiotic; ProbStim: experimental broiler diet with 0.01 % combination of probiotic and stimbiotic; SEM: Standard Error Mean.

## Funding and acknowledgment

The project was supported and funded by the Department of Animal Science, North- West University. The authors would like to express their gratitude to National Research Foundation, SA (NRF) and the North- West University Postgraduate funding programme.

## CRediT authorship contribution statement

**RM. Mokonyama:** Conceptualization, Data curation, Methodology, Investigation, Writing – original draft. **U. Marume:** Conceptualization, Funding acquisition, Resources, Supervision, Validation, Writing – review & editing. **Ghaneshree Moonsamy:** Conceptualization, Resources, Supervision, Validation.

## Disclosures

The authors hereby confirm that there are no conflict of interest in the submission of this manuscript.
